# Connecting foreign language enjoyment and English proficiency levels: The mediating role of L2 motivation

**DOI:** 10.3389/fpsyg.2023.1054657

**Published:** 2023-02-09

**Authors:** Haihua Wang, Lin Xu, Jiaxin Li

**Affiliations:** School of Foreign Languages, Dalian Maritime University, Dalian, China

**Keywords:** foreign language enjoyment, L2 motivation, language proficiency, foreign language learning, tertiary education

## Abstract

To further understand the connections between positive emotions, in particular foreign language enjoyment, second language (L2) motivation, and English achievement, the present study investigated how foreign language enjoyment and L2 motivation contribute to learners’ English achievement and the mediating role of motivation in the pathway. A questionnaire was employed to collect quantitative data from 512 university students learning English as a foreign language (EFL) in China. The results showed that the higher the language proficiency level, the higher the foreign language enjoyment level and the stronger the L2 motivation. Participants reported a significant difference in the private factor of foreign language enjoyment, the ideal L2 self, and the L2 learning experience among different language proficiency groups. Overall, foreign language enjoyment has a positive predictive effect on L2 motivation; however, the influence of different dimensions varies among language proficiency groups. Foreign language enjoyment is a positive predictor of English achievement, and motivation partially mediates this pathway. These findings provided an in-depth profile of foreign language enjoyment and L2 motivation of Chinese EFL learners at different language proficiency levels, highlighting the connections between positive emotion, motivation, and English achievement, and the contribution of foreign language enjoyment and L2 motivation in English learning. Based on these findings, pedagogical implications are suggested for English teaching and learning in Chinese tertiary education.

## 1. Introduction

“Language learning can be very emotional, as anyone who has ever tried to learn or use another language (L2) will attest” (Plonsky et al., 2022, p. 1). Indeed, research on emotions has a long history in the field of second language acquisition (SLA). However, the accumulated literature has exclusively focused on the negative affective factor: anxiety. The depth and breadth of research on this negative emotional factor in SLA are evident from the fact that Scovel (1978) reviewed research on anxiety as early as more than 40 years ago, and three decades later, Horwitz (2010) again combed through the timeline of theoretical and empirical research on anxiety. With the introduction of Positive Psychology (PP) into SLA (MacIntyre and Mercer, 2014), we see that the scope of emotion research in SLA is not limited to negative emotions; rather, it has a broader world. The full spectrum of emotions related to L2 development is under investigation now (Plonsky et al., 2022). PP emphasizes the positive aspects of an individual’s emotions and characteristics, which does not mean that negative emotions are ignored or even discarded in research, but rather aims to take a holistic perspective on human emotions. Riding on the wave of PP, L2 research on positive emotions is blooming and booming. A group of scholars, represented by MacIntyre and Dewaele, have done a sizeable amount of research on the role of positive emotional factors in L2 learning and performance from the PP perspective. The research topics address broad aspects of positive emotions, such as love (Pavelescu and Petrić, 2018), grit (Wei et al., 2020), and the most frequently discussed enjoyment (e.g., Dewaele and MacIntyre, 2014, 2016; Dewaele and Alfawzan, 2018; Jiang and Dewaele, 2019; Dewaele and Li, 2021; Tahmouresi and Papi, 2021; Li and Wei, 2022). The findings in these studies demonstrate that positive emotions play an important role in learners’ motivation, interest, performance, and achievement. The two basic theoretical frameworks that underpin the understanding of positive emotions in SLA are the broad-and-build theory (Fredrickson, 2001) and the control-value theory (Pekrun, 2006). The broad-and-build theory suggests that positive emotions could help learners’ effectiveness in absorbing input, thereby facilitating the L2 learning process (MacIntyre and Gregersen, 2012), and offsetting the effects of negative emotions. The control-value theory, on the other hand, proposes that learners’ perceived control of the task and the degree of valuing the outcome of the activity affect the students’ emotions during the learning process, and these emotions play a role in motivating learning. Both theories explain well the contribution of positive emotions to L2 performance.

The previous focus on developing learners’ cognitive abilities in L2 research, and the flourishing studies on positive emotions now, have fruitful results, but there is less exploration of L2 learners’ emotional factors and their interaction with motivational factors (Dewaele and Li, 2020). Positive emotions, in particular enjoyment, and motivation have been proved to have a positive effect on L2 performance (e.g., Saito et al., 2018; Jiang and Dewaele, 2019; Tahmouresi and Papi, 2021). Nevertheless, there is still uncertainty about the intrinsic connections between enjoyment, motivation, and language proficiency. To this end, deeply probing into this set of uncertainty might contribute to casting light on the interaction between positive emotion, motivation, and language proficiency. Using a sample of 512 Chinese tertiary students, the present study aims to profile the foreign language enjoyment and L2 motivation of EFL learners at different language proficiency levels, investigate the connection between foreign language enjoyment and L2 motivation, specifically the possible proficiency differences lying in the connection, and figure out the pathway by which enjoyment and motivation affect English achievement.

## 2. Review of literature

### 2.1. Foreign language enjoyment

As the positive counterpart of foreign language classroom anxiety (FLCA; Dewaele and MacIntyre, 2014), foreign language enjoyment (FLE) is becoming a popular topic in SLA emotion research. Enjoyment was broadly described as the feeling when people enjoy something, or simply the pleasure. Decades ago, Csikszentmihalyi (2004) made a distinction between two similar concepts: enjoyment and pleasure. Pleasure refers to the sense of satisfaction people experience when they believe that the standards set by biological programs or social training have been reached while enjoyment is when people’s abilities are stretched beyond what they previously believed they were capable of, or when they feel a sense of forwarding mobility and accomplishment. Dewaele and MacIntyre (2016) used an analogy to illustrate this pair of terms as playing games. It may be a pleasure to win a game, while one may feel enjoyable even losing a game if “one has performed better than expected against a superior opponent” (p. 217), revealing that enjoyment is related to both the process and the outcome. When it is related to the outcome, it tends to be more self-related. Later on, Teimouri (2017) specified such a positive emotion in the L2 research realm as “language learners experience in the process of learning or using the target language either within the boundary of a specific instructional context or in authentic real-life situations” (p. 689).

It needs to be clarified that the shift in the focus of research on emotional factors influencing EFL learning does not mean a trade-off; rather, it means a shift from the particular focus on anxiety (MacIntyre and Gregersen, 2012; Li et al., 2018) to “the full spectrum of emotions investigated in relation to L2 development” (Plonsky et al., 2022, p. 1). The last decade has seen the emergence of a large number of studies that examine both positive and negative emotions (e.g., MacIntyre and Gregersen, 2012; Dewaele and MacIntyre, 2014, 2016; Dewaele et al., 2017, 2019b; Boudreau et al., 2018; Reeve et al., 2018; Dewaele and Li, 2021; Li et al., 2021; Tahmouresi and Papi, 2021; Li and Wei, 2022). Among those, the study conducted by Dewaele and MacIntyre (2014) stands out since it was probably the first one to research on FLE in the L2 context and the effects of two emotional factors, enjoyment and anxiety, on L2 learners and the learning process were also carefully depicted. In their study, 1,746 foreign language learners were enrolled with a wide range of demographic backgrounds (sex, age, education, language being studied, foreign language mastery, relative standing in peers, nationality, and languages acquired). The results revealed that FLE and FLCA were negatively correlated, but “enjoyment and anxiety appear to be independent emotions, and not opposite ends of the same dimension” (p. 261). Participants reported significantly more enjoyment than anxiety in L2 classes. Of note, compared with males, the female participants scored higher on both FLE and FLCA. Moreover, higher levels of FLE were linked to multi-languages, relative standing in peers, higher foreign language proficiency, older age, and learning experience in tertiary education. In addition, North American learners showed the highest level of FLE, and Asian participants reported the opposite pattern. Furthermore, they conducted an in-depth investigation into the factors that make students feel enjoyable in foreign language classes. Through open-ended questions, participants gave feedback that supportive peers, teachers’ humor and encouragement, and creative classroom activities were the main sources of learning enjoyment.

Since then, studies have been conducted on FLE, as well as on the connections between FLE and other emotional and learner factors. Connecting FLE and learner variables, research was carried out with different demographic variables (e.g., Dewaele and MacIntyre, 2014, 2016) and various populations, including but not limited to Chinese secondary and tertiary EFL learners (e.g., Li, 2019; Li et al., 2019), Japanese high school students (Saito et al., 2018), British high school students (Dewaele et al., 2017), Romanian high school learners (Pavelescu and Petrić, 2018), British pupils and Saudi undergraduates (Dewaele and Alfawzan, 2018), Saudi Arabic university students (Alrabai, 2022), Spanish EFL learners (Dewaele et al., 2019b), and university students in Iran (Tahmouresi and Papi, 2021), etc. Considering the populations that have been mentioned above, even though differences exist, those studies clearly demonstrate the positive role of FLE in foreign language learning. One study worth mentioning was conducted by Dewey et al. (2018), because first, the research setting was a study-abroad program taking place in Jordan, and second, it was one of the few longitudinal studies (e.g., Dewaele and Dewaele, 2017; Elahi Shirvan and Taherian, 2018; Saito et al., 2018), as opposed to the majority of cross-sectional studies in this field. Similarly, in their 14 weeks study, they found learners grew more comfortable in the classroom when they experienced more enjoyment and less anxiety in the setting of studying abroad. The positive force was also found in the connection between FLE and experience abroad (Thompson and Lee, 2014), willingness to communicate in class (Khajavy et al., 2018; Saito et al., 2018), and emotional intelligence (Li and Xu, 2019). There are also in-depth studies looking at the intersection of FLE with cognitive factors and motivation (e.g., Teimouri, 2017; Papi and Khajavy, 2021), revealing the predictive role of motivation on FLE. The research mentioned above appeared to cover a diverse population in multiple aspects of emotion, but the actual width and depth of research on FLE are still limited compared to studies on anxiety, and some findings need further validation. Taking Chinese EFL learners as an example, Jiang and Dewaele (2019) suggest that Chinese EFL learners are to some degree “unique” compared to the international sample in Dewaele and MacIntyre (2014), who also found that FLE and FLCA coexist in foreign language learning and were significantly negatively correlated. This result was verified in a sample of 1,307 high school students in China (Li, 2019) but could not be generalized to the sample of foreign language learners aged 16–18 years in London (Dewaele and Dewaele, 2017). Therefore, FLE, as an emotional factor, that has only received attention in recent years needs to be further expounded in various contexts, including Chinese EFL learners, the largest EFL population in the world (You and Dörnyei, 2016), in order to verify and complement the existing research.

Among the aforementioned blossoming research themes, one research branch that must be mentioned is the conceptualization and measurement of FLE (e.g., Dewaele and MacIntyre, 2014, 2016; Li et al., 2018). In the pioneering study on FLE, Dewaele and MacIntyre (2014) adapted seven items from Ryan et al.’s (1990) interest/enjoyment subscale and developed the *Foreign Language Enjoyment Scale* with 21 items. Later on, they refined this scale into 14 items and constructed a two-factor structure, i.e., FLE-Social and FLE-Private (Dewaele and MacIntyre, 2016). The scale continued to be modified and refined in the following study, resulting in three dimensions: FLE-Social, FLE-Private, and Peer-Controlled versus Teacher-Controlled positive (Dewaele and Dewaele, 2017). The important twist came in 2018 (Li et al., 2018) when an FLE scale based on data of Chinese EFL learners emerged. The Chinese-localized *Foreign Language Enjoyment Scale* contains 11 items in three dimensions: FLE-Private, FLE-Teacher, and FLE-Atmosphere, which was believed to better understand the conceptualization of Chinese EFL learners’ FLE. According to Li (2019), FLE-Private was explained as “positive feelings boosted by fun, accomplishments, and interesting things in EFL learning” (p. 5); FLE-Teacher could be summarized as the positive feeling from teachers’ “encouraging and supportive attitudes” (p. 5), and FLE-Atmosphere refers to the positive EFL learning atmosphere in which learners could feel enjoyable.

Regarding the fertile sources of FLE, research findings show that the teacher factor in the scale is the pivotal one (e.g., Dewaele et al., 2017; Jiang and Dewaele, 2019; Jiang, 2020). For instance, the study conducted by Dewaele et al. (2019b) with Spanish EFL learners reported that teacher characteristics predict stronger FLE than FLCA, and teachers’ friendly behavior is the strongest positive predictor of FLE. In the same vein, Jiang and Dewaele (2019) carried out a study to explore the enjoyment and anxiety of Chinese EFL learners. Results indicated that FLE was more likely to be triggered by teachers. Additionally, students’ motivation and attitude toward the teacher are all positively correlated with students’ perception of the teachers’ happiness (Moskowitz and Dewaele, 2021). Considering the existing studies, it seems that teachers’ characteristics, as well as the class environment they foster, could have an effect on FLE. Teachers’ friendliness, encouragement, support, and not-too-strict requirements, along with the relaxed, joyful, and creative class environment will boost learners’ FLE.

The evidence presented thus far indicates that FLE has a positive influence on L2 learners and the L2 learning process. It has been found that FLE is significantly and positively correlated to L2 achievement (e.g., Saito et al., 2018; Jiang and Dewaele, 2019; Tahmouresi and Papi, 2021). For example, the findings in the study by Saito et al. (2018) revealed a significant relationship between FLE, motivation, and L2 speech performance. Dong and Liu (2022) attribute the positive effect of FLE to two main reasons: firstly, FLE can influence learners’ language learning behaviors, such as maintaining L2 learning engagement (Saito et al., 2018), regulating L2 learning strategies (Shao et al., 2019), and continuing L2 learning process (Dewaele et al., 2019b); secondly, FLE can also act on motivation (MacIntyre et al., 2019), and enhance learners’ L2 self-expectations (Shao et al., 2020).

Taken together these studies reviewed so far provide important insights into FLE as a typical positive emotion in various contexts. The evidence seems to suggest a relationship between FLE and other emotions, learners’ internal and external variables, and the learning process and performance. Unfortunately, there remain several aspects of how FLE impacts L2 motivation, and whether FLE of learners at different proficiency levels differs, which is unclear and not thoroughly explicated.

### 2.2. L2 motivation

Motivation is one of the frequently discussed learner variables in SLA literature. Ellis (1994) described L2 motivation as “the effort that learners put into learning the L2 as a result of their need or desire to learn it” (p. 504). Through ongoing efforts, L2 motivation researchers have developed several theoretical frameworks (e.g., Deci and Ryan, 1985; Gardner, 1985; Higgins, 1987; Dörnyei, 2009) to profile the motivation of EFL learners. Nourished from the fertile ground of SLA and psychology, the L2 motivation self-system (L2MSS), an integrative synthesis of L2 motivation constructs and approaches, “represents a major reformation of previous motivational thinking by its explicit utilization of psychological theories of the self” (Dörnyei, 2009, p. 9). It consists of three principal dimensions: the ideal L2 self (IL2S), the ought-to L2 self (OL2S), and the L2 learning experience (L2LE). IL2S is “the L2-specific facet of one’s ‘ideal self’” (Dörnyei, 2009, p. 29), referring to the image L2 learners imagine themselves to be when they complete their studies and become successful learners. It is usually considered the intrinsic motivational factor of L2 learners. For example, when they study abroad, they would be able to comprehend lectures in English, understand English movies, and communicate with foreign friends effectively. However, there is a gap between the real self and the imagined self sometimes, and it is where motivation comes in. OL2S is defined as “the attributes that one believes one ought to possess to meet expectations and to avoid possible negative outcomes” (Dörnyei, 2009, p. 29). In contrast to IL2S, this dimension can be understood as the external factors that influence the learner’s motivation, such as the desire to meet parental expectations, and not to disappoint the teacher. L2LE refers to “‘executive’ motives related to the immediate learning environment and experience” (Dörnyei, 2009, p. 29), such factors as the teacher, the peers, the curriculum, or the experience of success.

It is believed that motivation is linked to “an individual’s personal core, forming an important part of one’s identity” (Dörnyei, 2009, p. 9). Thus, using this theoretical model, a large number of studies have been conducted on different populations (e.g., Taguchi et al., 2009; Csizér and Lukács, 2010; Lamb, 2012; Islam et al., 2013; You and Dörnyei, 2016; Teimouri, 2017; Saito et al., 2018; Papi and Khajavy, 2021). These studies not only documented L2 learners’ motivation characteristics in different contexts but also tested the validation and adaptiveness of the framework. Under the L2MSS framework, previous studies showed consistent results indicating that compared with the weak effect of OL2S, IL2S and L2LE shared a stronger predictive power (e.g., Taguchi et al., 2009; Papi, 2010; Papi and Teimouri, 2012, 2014; You and Dörnyei, 2016; Teimouri, 2017; Wong, 2020; Papi and Khajavy, 2021).

There are now studies that have applied the framework in research on Chinese EFL learners (e.g., Magid, 2009; Taguchi et al., 2009; Li, 2014), notably among which is the study carried out by You and Dörnyei (2016). They conducted a large-scale survey of 10,000 Chinese EFL learners. The sample was stratified according to geographical region (eastern, central, and western), teaching context (secondary school with a sub-category of urban and rural, and university with two sub-categories of key and ordinary, English and non-English majors). Some main findings are: (1) Chinese learners tend to have positive ideal self-images with English; (2) Chinese learners are not primarily instrumentally motivated as widely believed; (3) OL2S scores lowest; (4) female L2 learners score significantly higher than male learners; (5) there is an east–west disparity because of economic stratification; and (6) the more advanced or specialized one’s education, the stronger ideal image the student entertains. These findings provided important information for better understanding the motivation levels and characteristics of Chinese EFL learners and for comparing motivational characteristics with those of learners in different contexts.

In addition to the research focus on L2 learners’ motivation levels and characteristics, some researchers have explored the relationship between motivation and other learner factors, such as emotional factors, and academic achievement. Existing research noted that students’ higher levels of motivation and FLE are linked to improved performance in L2 achievement (e.g., Saito et al., 2018; Cocca and Cocca, 2019; Yu, 2019; Tahmouresi and Papi, 2021). Furthermore, much of the available literature reviewed until now not only demonstrates the positive effects of motivation and enjoyment but also points to the close relationship between these two variables. For example, Méndez-Aguado et al. (2020) examined the influence of emotions on motivation and performance with a sample of students learning French as a foreign language. Their findings indicated that positive emotions (e.g., enjoyment and pride) are positive predictors of motivation, while negative emotions (e.g., anxiety and boredom) are negative ones. The findings of Papi (2010) revealed that IL2S negatively but OL2S positively predicts anxiety; similarly, Teimouri (2017) found a positive relationship between IL2S and joy, whereas OL2S relates to anxiety. Moreover, further pathways have been explored regarding FLE, motivation, and learning achievement. Using a sample of 335 English major students in China, Zhang et al. (2020) proved the positive influence of motivation on language learning and FLE is an effective path between motivational orientations and L2 proficiency. Nonetheless, only a small number of studies have focused on the mediating role of motivation, among which Dong and Liu (2022) examined the effects of FLE on L2 performance and the mediating role of motivation. Their findings reveal that FLE significantly positively predicts foreign language test performance both through a direct pathway and indirectly *via* learners’ L2 use and expectancy component of motivation. Despite the achievements of previous studies, the connection between FLE and L2 motivation and the mediating role of L2 motivation still need further investigation.

### 2.3. English proficiency test

The College English Test (CET) is a nationwide standardized examination aiming at promoting the teaching of English at universities in China, and providing an objective and accurate measurement of the English language proficiency of university students. Beginning in 1987, the CET, which consists of Band 4 (CET-4) and Band 6 (CET-6), has gone through about 35 years. It is a norm-referenced and criteria-related proficiency test, with high reliability and validity, and a positive backwash effect on English teaching at the tertiary level in China. The norm-referenced scores from 220 to 710 are reported in CET-4 and CET-6 tests. To enrich the interpretation of test scores, the National College English Testing Committee conducted an alignment study to link the CET scores to the Common European Framework of Reference for Language (CEFR; Council of Europe, 2020). CEFR describes the linguistic, sociolinguistic, and pragmatic abilities required for language learners or users to complete communicative activities, and categorizes language learners into three broad levels: basic users (A1, A2), independent users (B1, B2), and proficient users (C1, C2). International and regional exams such as TOEFL (Tannenbaum and Wylie, 2008), and IELTS (Taylor, 2004), have been aligned with the CEFR to make the tests more widely recognized. Jin et al. (2022) presented the rationale for the alignment study and reported the findings: with a reported score of 388 in CET-4, students could meet the minimum language proficiency requirements for CEFR B1 and 549 for CEFR B2, and with a total score of 438 in CET-6, students could meet the minimum requirements for CEFR B2 and 596 for CEFR C1. This study realized the alignment of the CET with international language proficiency standards, making the CET scores referenceable to international standards.

### 2.4. Objectives of the study

In light of the review above, it could be seen that neither the dimensions of FLE nor the L2MSS were depicted with adequate clarity in the context of Chinese tertiary education. Moreover, the relationship between FLE as a typical positive emotional factor and L2 motivation as one of the frequently researched learner variables is still under-researched, especially when taking the difference among various English proficiency levels into consideration. In addition, most of the reviewed L2 research focused exclusively on how emotions, positive or negative, influence L2 learning performance, leaving behind the synergistic role of other factors, such as motivation in the process. Accordingly, the paucity of research on the mediating role of motivation deserves our efforts to further expound on the close connections between FLE, L2 motivation, and English achievement.

To address these issues, the present study examines whether we can extend connections between FLE and L2 motivation to English achievement and delves into how FLE can affect L2 motivation and academic performance, especially the different degrees of influence among proficiency levels. The current study is intended to answer the following research questions:

1. What levels of FLE and L2 motivation do Chinese tertiary EFL learners report? Are there proficiency differences in terms of their FLE and L2 motivation?
2. How are FLE and L2 motivation related to each other among Chinese EFL learners at four different proficiency levels?
3. How is the relationship between FLE and English achievement mediated by the three dimensions of L2MSS?

## 3. Methods

### 3.1. Participants

The present study applied convenience sampling. Altogether, 512 Chinese non-English majors from a university in northeastern China completed the online questionnaire. The respondents consisted of 344 males (67.2%) and 168 females (32.8%), aged between 18 and 20 years. All of the participants were admitted to the university *via* the National College Entrance Examination and thus had a comparable amount of English learning experience. At the time of sampling, all the participants were enrolled in the required course for non-English majors, namely *College English*, designed to help college students enhance their overall English skills required in *College English Teaching Guidelines* (National College Foreign Language Teaching Advisory Board, 2020). They had taken the CET-4 and got the score reports.

### 3.2. Instruments

A composite questionnaire with two sections was used as the major instrument in the present study. For a better understanding, the questionnaire was written in Chinese. The first section was composed of items about the participants’ background information including gender, grade, and scores of CET-4. The second section was comprised of 27 items measuring the participants’ L2 motivation and enjoyment concerning English learning. All the items in this section were on a five-point Likert scale, ranging from 1 (*strongly disagree*) to 5 (*strongly agree*). The subscales used in the questionnaire are described in full below.

### 3.3. FLE measurement

To measure the participants’ FLE, nine items in the questionnaire were selected from *the Chinese version of Foreign Language Enjoyment Scale* (CFLES) adapted by Li et al. (2018) from the original *Foreign Language Enjoyment Scale* (Dewaele and MacIntyre, 2014). Three dimensions, namely FLE-Private, FLE-Teacher, and FLE-Atmosphere with three items in each were used to assess the participants’ enjoyment of learning English. The scale analysis of the nine items on FLE measurement revealed high internal consistency (*Cronbach’s alpha* = 0.938). The KMO value and *p*-value of Bartlett’s spherical test are 0.910 and 0.000, indicating that the CFLES has high validity.

### 3.4. L2 motivation measurement

On the basis of *the L2MSS Questionnaire* by Papi (2010) who developed the questionnaire by referring to Dörnyei’s guidelines (2003), 18 items in the questionnaire were selected. Six items in each of the three dimensions (IL2S, OL2S, and L2LE) were used to measure the participants’ L2 motivation. Some of the items were slightly modified to better fit the local context. High internal consistency was found in the scale analysis of the 18 L2 motivation measuring items (*Cronbach’s alpha* = 0.943). The KMO value and *p*-value of Bartlett’s spherical test are 0.949 and 0.000, suggesting that the L2MSS Questionnaire is of high validity.

### 3.5. English proficiency measurement

In the present research, CET-4 scores were used to determine the participants’ English proficiency levels. No passing line was established for CET-4, and norm-referenced scores (220–710) were reported. The norm group for CET-4 was selected from ~30,000 non-English major test takers from 16 colleges and universities nationwide. By referring to the norm, the raw scores were converted into reported scores. The total reported score for CET-4 is 710, calculated as follows^1^:


$$\text{TotSco}=\frac{\left(X-\text{Mean}\right)}{SD}\times 70+500$$

In the formula, TotSco means the total score; X means the raw total score of each test taker. CET-4 raw scores of test takers are converted to reported scores by referring to this norm formula. A percentile rank in the norm population is assigned to each candidate’s reported score. For example, a candidate with a total CET-4 reported score of 450 has a corresponding percentile position in the norm group of 25%, indicating that this candidate outperforms 25% of the norm group in English but is inferior to 75% of the norm group. Plotting CET-4 scores as a normal distribution, the standardized score intervals of CET-4 scores are below 360, 361–430, 431–500, 501–570, 571–640, and above 641. Based on the percentile position of the test takers, the participants in this study were divided into four groups: Group 1, elementary level (220–430 scores, *n* = 91, *m* = 407.91, SD = 24.319); Group 2, lower intermediate level (431–500 scores, *n* = 248, *m* = 465.11, SD = 20.777); Group 3, upper intermediate level (501–570 scores, *n* = 138, *m* = 530.49, SD = 19.798); and Group 4, advanced level (571–710 scores, *n* = 35, *m* = 595.31, SD = 21.565).

### 3.6. Procedure

The composite questionnaire was piloted among 44 Chinese EFL learners who made up the peer group for the study participants, and the questionnaire was modified in response to their feedback. With the course instructors’ approval, the online questionnaire survey was conducted between April 2022 and May 2022. The students were reassured to participate in the survey voluntarily and informed of the purpose of this anonymous data collection. The online questionnaire was administered to the participants during the intermission between class times.

### 3.7. Data analysis

A total of 554 responses were collected, of which 512 were valid (92.42%). The collected data were then entered into SPSS (version 26.0) for statistical analyses. The descriptive statistical analysis was carried out on all the variables in the survey including mean, standard deviation, and min and max values. Then, Cronbach’s alpha coefficients were used to determine the internal consistency of the motivational and emotional variables as well as each dimension in the subscales. A value of α coefficient higher than 0.8 indicates high reliability. A value between 0.7 and 0.8 indicates good reliability. A value between 0.6 and 0.7 indicates acceptable reliability, and a value <0.6 indicates poor reliability (Eisinga et al., 2013). Finally, PROCESS v2.16.3 was run to measure the direct and indirect effect of emotional and motivational variables on English achievement, and a series of variance, correlation, multiple regression, and interaction analyses were done to address the research questions.

## 4. Results

### 4.1. FLE and L2 motivation of Chinese tertiary EFL learners

Descriptive statistics of FLE and L2 motivation of the Chinese undergraduates at different English proficiency levels are shown in Table 1, revealing the enjoyment and motivation of Chinese tertiary EFL learners in several aspects. The mean value of FLE as a whole is 3.75, and the mean score of FLE-Teacher is the highest (*m* = 4.07), followed by FLE-Atmosphere (*m* = 3.87) and FLE-Private (*m* = 3.30). As for L2 motivation, the mean value is 3.60. A closer inspection of the table shows the sequence of mean values of dimensions in L2MSS from the lowest to the highest is OL2S (*m* = 3.49), L2LE (*m* = 3.53), IL2S (*m* = 3.79).

**Table 1:** Descriptive statistics of FLE and L2MSS.

	Min	Max	Mean	SD	Skewness	Kurtosis
FLE-Private	1.00	5.00	3.30	1.04	−0.221	−0.219
FLE-Teacher	1.00	5.00	4.07	0.96	−1.037	0.840
FLE-Atmosphere	1.00	5.00	3.87	1.01	−0.708	0.099
FLE	1.00	5.00	3.75	0.90	−0.748	0.594
IL2S	1.00	5.00	3.79	1.01	−0.738	0.300
OL2S	1.00	5.00	3.49	0.98	−0.349	−0.099
L2LE	1.00	5.00	3.53	1.034	−0.457	−0.088
L2MSS	1.00	5.00	3.60	0.90	−0.526	0.499

Furthermore, it is apparent from Table 2 that the mean values of FLE and L2 motivation increase with English proficiency levels. Standard deviation is the quantitative indicator to reflect the degree of dispersion of a set of data. Data in Table 2 demonstrates the mean value of FLE (*m* = 4.19) and L2MSS (*m* = 3.86) in Group 4, the advanced learners, is higher than in other groups, while the standard deviation is relatively lower (FLE, SD = 0.67; L2MSS, SD = 0.81), indicating that L2 learners in Group 4 are more enjoyable and better motivated in EFL learning, and at the same time, their performance in FLE and L2 motivation is relatively more stable than those in the other groups. By contrast, L2 learners in Group 1 share the lowest mean value and relatively small standard deviation, meaning that the learners at the elementary proficiency level have less FLE and weaker L2 motivation in EFL learning. Compared with Group 4 and Group 1, standard deviations of FLE (0.95, 0.83) and L2MSS (0.93, 0.79) in Group 2 and Group 3 indicate great variations among L2 learners in their enjoyment and motivation in English learning, with the broad range in Max. and Min. value pointing to the huge individual differences between L2 learners.

**Table 2:** Descriptive statistics of FLE and L2MSS at different proficiency levels.

	FLE				L2MSS			
	Min	Max	Mean	SD	Min	Max	Mean	SD
Group1	0.89	4.44	3.07	0.74	1.00	5.00	3.40	0.95
Group2	1.00	5.00	3.70	0.95	1.00	5.00	3.55	0.93
Group3	1.00	5.00	3.89	0.83	1.00	5.00	3.77	0.79
Group4	2.67	5.00	4.19	0.67	2.22	5.00	3.86	0.81

The results of one-way ANOVA revealed a significant difference (*p* < 0.05) among four groups at different English proficiency levels regarding FLE and L2 motivation, specifically in dimensions of FLE-Private, IL2S, L2LE, whereas in dimensions of FLE-Teacher (*p* = 0.090), FLE-Atmosphere (*p* = 0.509), and OL2S (*p* = 0.588), a statistically significant difference between groups was not found.

*Post hoc* tests showed that the FLE enhanced as the English proficiency level developed, and multiple comparisons indicated a stepwise increase in the difference between groups. A significant difference was found with a small-to-medium effect size (Plonsky and Oswald, 2014) between Group 4 and Group 1 (*d* = 0.71*), between Group 3 and Group 2 (*d* = 0.19*) respectively, and a subtle but not statistically significant difference between Group 3 and Group 4 (*d* = 0.30). Differences in the dimensions within the FLE varied among groups at different English proficiency levels. In terms of FLE-Private, the results of *post hoc* multiple comparisons between the four groups showed significant differences (*p* < 0.05). Significant differences with a small effect size were found between Group 1 and Group 2, Group 2 and Group 3, and Group 3 and Group 4 (*d* = 0.46*, 0.45*, 0.47*). The most significant difference was found between Group 1 and Group 4 with a large effect size (*d* = 1.37*). The differences between the four groups in terms of FLE-Teacher were small. Significant differences were found only with a small effect size between Group 1 and Group 4 (*d* = 0.46*) and between Group 2 and Group 4 (*d* = 0.35*), respectively. FLE-Atmosphere differed least among the four groups compared to the two dimensions mentioned above, and *post hoc* multiple comparison results showed no significant differences.

Regarding L2 motivation, a different scenario is revealed. Although the overall trend was similar to FLE, the results of the multiple comparisons showed significant differences in L2MSS with a small effect size between Group 1 and Group 3 (*d* = 0.37*), Group 1 and Group 4 (*d* = 0.46*), and Group 2 and Group 3(*d* = 0.22*). There are slight differences in L2MSS between Group 1 and Group 2 (*d* = 0.15), Group 2 and Group 4 (*d* = 0.31), and Group 4 and Group 3 (*d* = 0.08), but not statistically significant. The three dimensions within L2MSS present different situations among the four groups. The mean difference of OL2S among four groups at different English proficiency levels was not statistically significant (*p* < 0.05), although they differed. For IL2S, only the difference between Groups 3 and Group 4 (*d* = 0.22) is not significant, while all other groups showed significant differences with a small-to-medium effect size; the largest difference lies between Groups 1 and Group 4 (*d* = 0.73*). The differences in L2LE between the two groups with similar scores were not significant: Group 1 and Group 2 (*d* = 0.18), Group 3 and Group 4 (*d* = 0.21); the differences between the other groups were statistically significant with a small-to-medium effect size. Similar to the other dimensions in FLE and L2MSS, the largest difference existed between Groups 1 and Group 4 (*d* = 0.71*).

### 4.2. Relationship between FLE and L2 motivation at different English proficiency levels

A Spearman correlation analysis was performed to investigate the relationship strength among the dimensions of FLE and L2 motivation after a normal distribution test. The results showed significant positive correlations (*p* <0.01) between FLE and L2 motivation overall and across dimensions among the four groups.

Multiple regression analyses were run with FLE-Private, FLE-Teacher, and FLE-Atmosphere as predictors, and L2 motivation as the outcome to investigate to what degree FLE could predict L2 learners’ motivation. As listed in Table 3, FLE positively predicts L2 learners’ motivation at different English proficiency levels. The coefficients of the models show a good model fit: Group 1 (*R* = 0.766, *R^2^* = 0.587, *F* = 41.273**), Group 2 (*R* = 0.841, *R^2^* = 0.707, *F* = 196.249**), Group 3 (*R* = 0.804, *R^2^* = 0.647, *F* = 81.923**), and Group 4 (*R* = 0.746, *R^2^* = 0.557, *F* = 12.971**).

**Table 3:** Multiple regression analysis on FLE and L2 motivation at different proficiency levels.

	Regression equations		Fit index			Coefficient	
	Predictor	Outcome	*R*	*R* ^2^	*F*	Value of *p*	*t*
Group1	FLE-Private	L2MSS	0.766	0.587	41.273^**^	0.014	2.496
	FLE-Teacher					0.942	−0.072
	FLE-Atmosphere					0.000	4.696
Group2	FLE-Private	L2MSS	0.841	0.707	196.249^**^	0.000	7.957
	FLE-Teacher					0.006	2.772
	FLE-Atmosphere					0.000	3.599
Group3	FLE-Private	L2MSS	0.804	0.647	81.923^**^	0.000	5.248
	FLE-Teacher					0.890	0.139
	FLE-Atmosphere					0.000	4.116
Group4	FLE-Private	L2MSS	0.746	0.557	12.971^**^	0.037	2.179
	FLE-Teacher					0.016	2.543
	FLE-Atmosphere					0.655	0.451

** *p* < 0.01.

**Figure 1:**
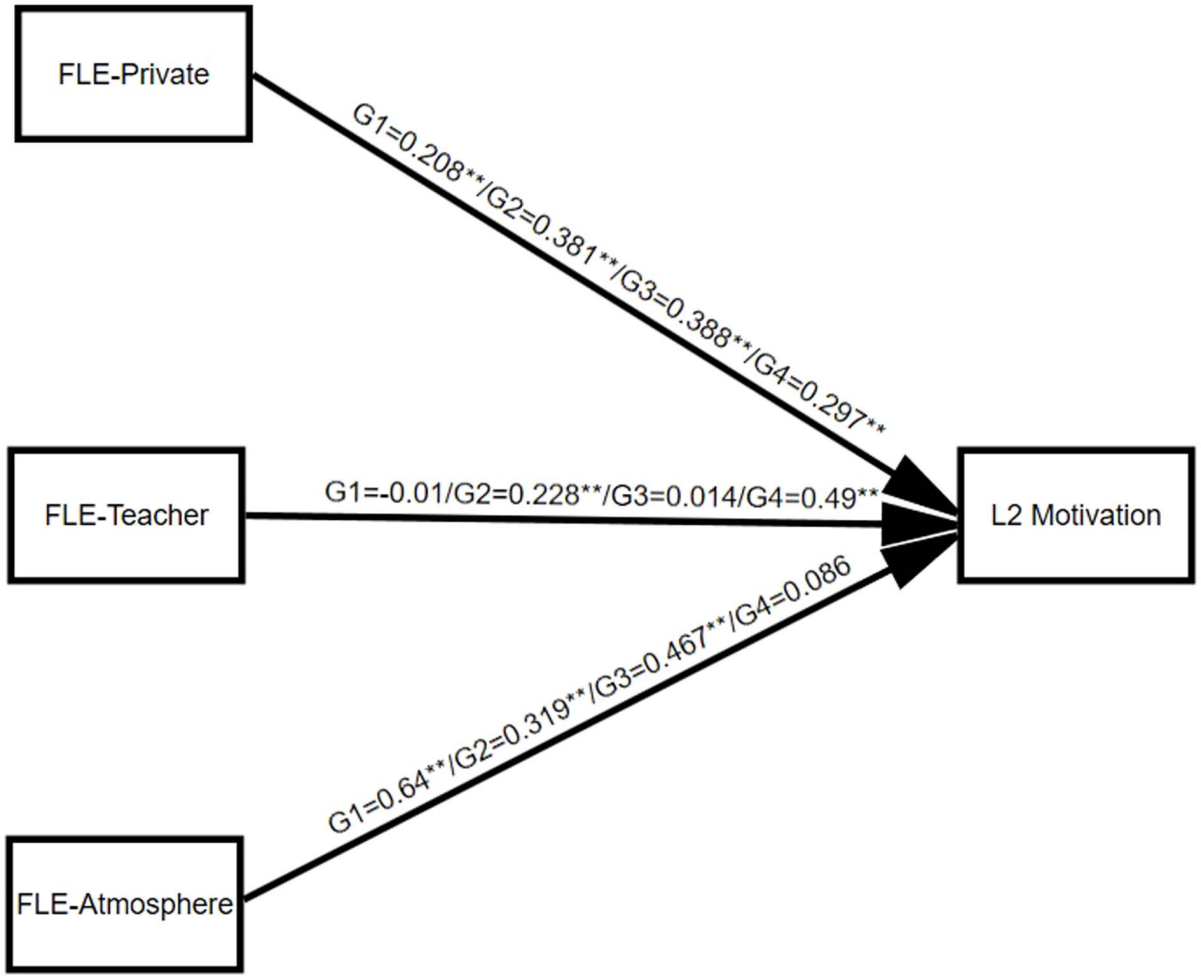
G1 = Group 1, G2 = Group 2, G3 = Group 3, G4 = Group 4, ** *p* <0.01.

As shown in Figure 1, FLE-Private positively and significantly predicted L2 motivation in four groups. Of the four groups, FLE-Private in Group 3 exerts the strongest influence over L2 motivation (*β* = 0.388**). FLE-Teacher significantly and positively predicted L2 motivation in Group 2 (*β* = 0.228**) and Group 4 (*β* = 0.490**). However, the effect of FLE-Teacher on L2 learners’ motivation was reduced to a non-significant level in Group 1 (*β* = -0.01) and Group 3 (*β* = 0.014). As for the dimension of FLE-Atmosphere, there is a significant positive prediction of L2 learners’ motivation in Groups 1, 2, and 3. The motivation of L2 learners in Group 1 (*β* = 0.640**) was most influenced by their enjoyment of the learning atmosphere; likewise, the effect of FLE-Atmosphere on motivation in Group 4 (*β* = 0.086) decreased to a non-significant level.

### 4.3. Mediating role of L2 motivation

Mediation testing was conducted by using a series of correlation and regression analyses under the guidelines of Baron and Kenny (1986). As presented in Table 4, variables of FLE, L2MSS, and English achievement are strongly correlated except for OL2S and English achievement (*r* = −0.010, *p* = 0.818). The results show that L2 learners’ FLE influenced their English achievement positively and significantly (*r* = 0.189, *p* < 0.01) as well as their IL2S, OL2S, and L2LE (*r* = 0.765, 0.576, 0.815, *p* < 0.01). Even after controlling for the effect of FLE on English achievement, IL2S, OL2S, and L2LE have significant effects on English achievement. According to conditions of mediation (Baron and Kenny, 1986), these results indicate that L2 motivation had a partial mediating effect on the relationship between learners’ FLE and English achievement (adjusted *R^2^* = 0.832, *p* < 0.001).

**Table 4:** Correlation analyses on FLE, L2MSS, and English achievement.

	1	2	3	4	5	6
FLE	—	0.765^**^	0.576^**^	0.815^**^	0.808^**^	0.189^**^
IL2S	0.765^**^	—	0.651^**^	0.752^**^	0.899^**^	0.171^**^
OL2S	0.576^**^	0.651^**^	—	0.674^**^	0.865^**^	−0.010
L2LE	0.815^**^	0.752^**^	0.674^**^	—	0.910^**^	0.191^**^
L2MSS	0.808^**^	0.899^**^	0.865^**^	0.910^**^	—	0.134^**^
EA	0.189^**^	0.171^**^	−0.010	0.191^**^	0.134^**^	—

** *p* < 0.01.

**Figure 2:**
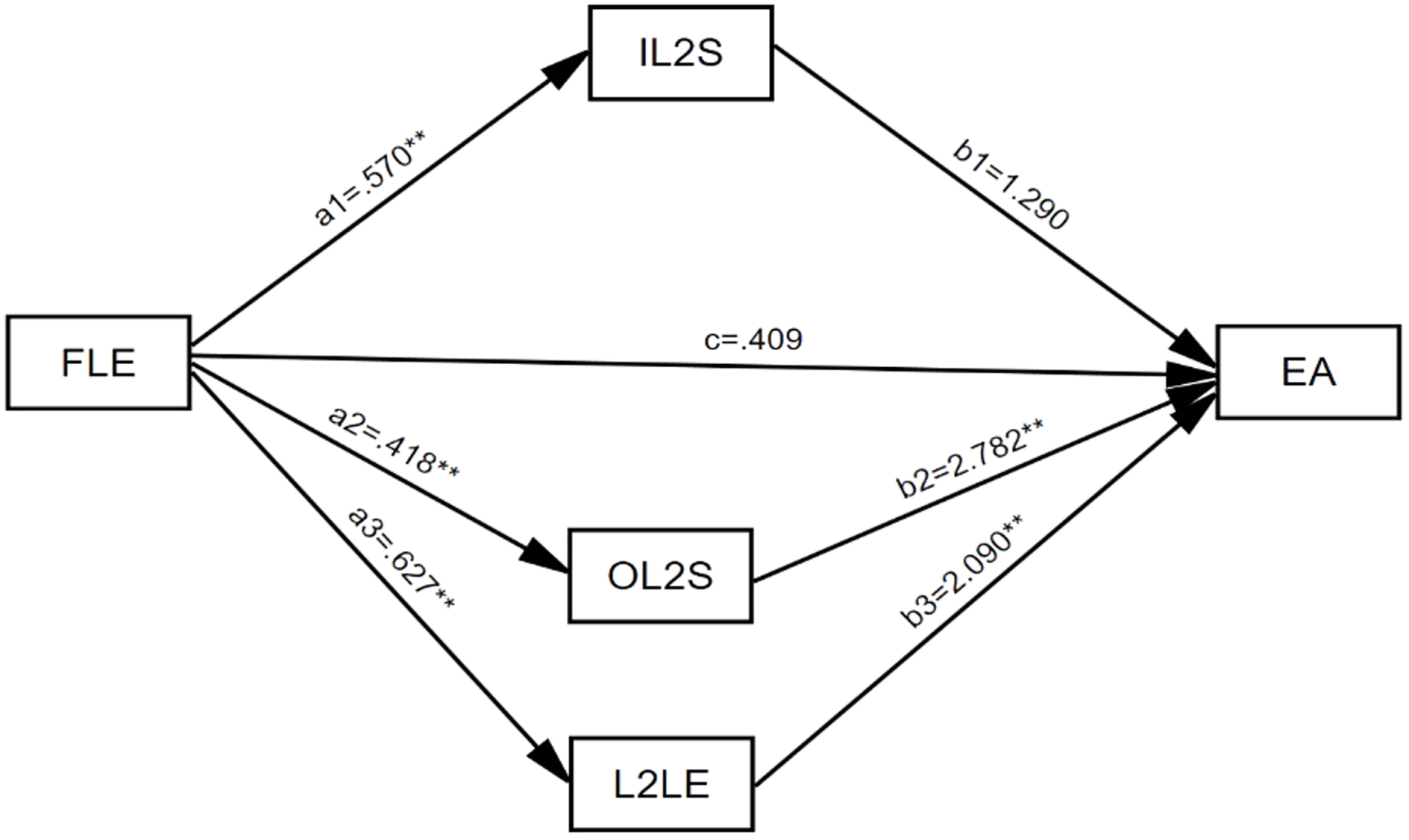
** *p* ≤ 0.01, EA, English achievement.

The mediator model was visualized in Figure 2. When IL2S, OL2S, and L2LE entered the regression equation model as the common predictor of English achievement, the coefficients of the model (*R* = 0.288, *R^2^* = 0.083, *F* = 11.497, *p* < *0*.001) showed that the model fit was acceptable. The multicollinearity diagnosis results demonstrate that the VIFs are all <2, and the possibility of collinearity problems between variables is extremely small.

Using PROCESS v2.16.3 (Model 4) to calculate the mediating effect of each path, the output is presented in Table 5. The direct effect is obtained as 0.409. Among the three mediating factors of L2MSS, L2LE has the biggest effect size (1.310), followed by OL2S (−1.163), while IL2S has the lowest effect size (0.735). The 95% confidence intervals for the above three paths, except for IL2S, do not include 0, indicating all of them reach the significant level. It can be seen that IL2S, OL2S, and L2LE play multiple parallel mediating roles between FLE and English achievement.

**Table 5:** Effect size and significance of each path.

Pathway	Effect size	SE	BCa 95% CI
FLE-IL2S-EA	0.735	0.676	[−0.0379, 2.618]
FLE-OL2S-EA	1.163	0.571	[−3.9034, −1.6611]
FLE-L2LE-EA	1.310	0.740	[0.6358, 3.5447]
FLE-EA	0.409	0.549	[−0.6694, 1.4877]

## 5. Discussion

This study set out with the aim of detecting the connection between Chinese tertiary EFL learners’ FLE and English proficiency with L2 motivation as a mediator. On the overall level of FLE, the mean value in the present study is slightly lower than the mean value reported by the previous study with Chinese EFL learners at the tertiary level (Jiang and Dewaele, 2019), and higher than the study with students in high school (Li et al., 2018). The relieved pressure after entering higher education may contribute to the increase in the level of enjoyment of university students. Another important finding in enjoyment is that teacher factors have a greater impact on students’ FLE than other factors. It is encouraging to compare this finding with that of Li et al.’s (2018) which claimed that high school students scored highest on the dimension FLE-Teacher, followed by FLE-Private and FLE-Atmosphere. Although the latter two factors are in a different order in the present study, the importance of the teacher factors is evident and in line with previous studies (e.g., Dewaele et al., 2019b; Jiang and Dewaele, 2019; Jiang, 2020; Li et al., 2021). Nevertheless, this finding is contrary to the study by Ahmadi-Azad et al. (2020) about teachers’ personality traits on FLE which proved the FLE-Private factor as the most significant one for Iranian EFL learners than the teacher factor. Meanwhile, this study pointed out that among the big five personality factors of teachers, openness, extroversion, and agreeableness are conducive to EFL learners’ FLE, which may, to some extent, shed light on the interpretation of the results of the current study. The greatest variation in FLE across English proficiency levels lies in learners’ internal factors, while enjoyment due to external factors of teachers and atmosphere varied less, suggesting that, on the one hand, despite the greater influence of the teacher factor on FLE, students at different language proficiency levels share a similar feeling; on the other hand, the degree to which learning itself can contribute to students’ FLE varies widely across different language proficiency levels.

As regards the overall level of L2 motivation reported by the participants, the relatively high mean value for IL2S echoes Tahmouresi and Papi’s results (2021) on L2 motivation in writing. Another finding is that the reported lowest mean value for OL2S is in line with the finding of a large-scale survey on language learning motivation in China (You and Dörnyei, 2016). It seems that Chinese L2 learners are more motivated by intrinsic rather than extrinsic factors, which is explained as a contradictory stereotype that Chinese learners are “less individualistic and more societally determined” (You and Dörnyei, 2016, p. 506). In addition, considering English proficiency, there is little difference among students across language proficiency levels in terms of the degree to which they are motivated by extrinsic factors. A notable difference exists in the intrinsic factor, that is IL2S, between the learners at the advanced and elementary proficiency levels. A possible explanation for this might be that successful learners are more likely to imagine their future image, whereas unsuccessful learners are less likely to be motivated by what the future will look like because they are farther removed from the goal of success. The difference between learners at the advanced and elementary proficiency levels is also greater in the degree to which learners are motivated by their learning experiences. The difference might be explained in part by the fact that students at the advanced proficiency level are easier to benefit from past enjoyable, effective, and successful learning experiences; conversely, the elementary proficiency level students’ past learning experiences do not play a positive role in motivation.

It is interesting to note that students at the advanced proficiency level are both similar to and different from students at the elementary proficiency level. The similarity lies in the fact that there is less individual variation in L2 motivation and FLE between these two groups of students, i.e., they are more similar to their peers in terms of motivation and enjoyment. The differences lie in the fact that most L2 learners at the advanced proficiency level have stronger motivation and more enjoyable learning experience, while students at the elementary level are less motivated and less likely to enjoy learning. Students at the intermediate levels vary more in both FLE and L2 motivation than students at either end of the spectrum. Thus, facilitating the learning process of students at such levels may be the main arena where teachers could play a role.

Succinctly stated, as the English proficiency level improves, the overall level of FLE rises, and motivation increases. These results corroborate those of previous studies (Vansteenkiste et al., 2009; Ryan and Deci, 2020; Howard et al., 2021; Li et al., 2022), indicating that students with a higher level of English proficiency share the trait of feeling more enjoyable in the learning process and are more motivated to learn, and vice versa.

With respect to the second research question, the current study demonstrated that FLE positively predicts L2 learners’ motivation at different language proficiency levels, although the strength differs. This finding accords with earlier observations on the association between enjoyment and motivation (Teimouri, 2017; Papi et al., 2019; Papi and Khajavy, 2021; Tahmouresi and Papi, 2021). The possible explanation is that learning enjoyment as a positive, activating, and activity-related achievement emotion (Pekrun, 2006) has been linked to the most intrinsic motivational types and levels (Noels et al., 2003).

Taking a closer look at the relationship between FLE and L2 motivation, we found that the teacher factor is the most powerful predictor of L2 motivation, and the atmosphere factor is the weakest for the students at the advanced proficiency level. And it is surprising to find that the teacher factor is the weakest predictor of L2 motivation for students at the elementary proficiency level. The issue emerging from these findings might be the lack of teachers’ concern for unsuccessful students in EFL classrooms. It is somewhat at odds with our inherent teaching experience, which generally assumes that teachers are more inclined to encourage poor students and motivate them to learn. However, the results suggest that teachers in fact pay less attention to unsuccessful learners so that such learners receive less learning enjoyment from teachers to get them motivated. Even worse, the level of FLE from teachers is so low that it may even demotivate L2 learning to some extent. In contrast, successful learners receive more encouragement and recognition from teachers, which better stimulates their interest in learning and creates a virtuous circle. Hence, teachers should assign priority over poor language learners in EFL classrooms, be kinder to them, encourage them and recognize their progress, so as to better motivate them and achieve better teaching results.

Interestingly, regarding the atmosphere factor in FLE, students at the advanced and the elementary proficiency levels showed the opposite result from the teacher factor, namely, the atmosphere factor is the strongest predictor of motivation for unsuccessful language learners and the weakest for successful learners. It could be argued that the possible reason for this finding may be that successful learners are not sufficiently motivated by the level of enjoyment from the classroom atmosphere, the learning atmosphere of their surrounding peers, and the interaction style in class, meaning that good students are more likely to be intrinsically motivated. In contrast, poor language learners are more interested in the pleasures of the surrounding learning atmosphere, i.e., they are more likely to be influenced by the external environment. These findings raise intriguing questions regarding the validity of graded instruction. Previous studies (Khajavy et al., 2018; Xia and Xu, 2018; Li et al., 2021) proved that the classroom environment has profoundly predictive effects on learners’ emotions. Positive classroom environments tend to make L2 learners feel more at ease and enjoy learning more. If the learning atmosphere of the surrounding peers, or the classroom atmosphere is not satisfying, does it play a negative role in the learning enjoyment and even hinder the motivation of poor language learners? Definitely, we cannot conclude that the learning atmosphere of unsuccessful students must be discouraging, but we cannot deny that this possibility does exist. As such, a positive learning atmosphere created by peers might be more conducive to motivating poor language learners and promoting their learning outcomes. The private factor of FLE is a significant positive predictor of motivation, regardless of language proficiency difference. It seems that the intrinsic factor is the stable factor that motivates learning.

In summary, these findings broadly support the control-value theory (Pekrun, 2018), which suggests that positive emotions such as enjoyment are promoted when learners feel in control of their learning and value academic achievement, and such emotions could influence motivation, self-regulation, and external regulation of learning. As a result, these results here are conducive to understanding the relationship between learners’ emotional factors and motivation, as well as guiding future instruction.

Concerning the third research question, we found that FLE could significantly and positively influence English achievement, and motivation had a partial mediation effect on the relationship between FLE and English achievement. The finding of the positive effect of enjoyment is in line with those of recent studies on academic performance and achievement within the socio-educational context of China: on junior secondary students (Li and Wei, 2022;), on secondary students (Dong and Liu, 2022), on undergraduate students in online classes (Li and Han, 2022), and some others (e.g., Dewaele and Alfawzan, 2018; Jiang and Dewaele, 2019). Positive academic emotions can increase learners’ resilience, thus contributing to academic performance and achievement (Dewaele et al., 2019a; Shao et al., 2020). FLE as a positive academic emotion could possibly help learners by counteracting the negative effects of negative emotions and facilitating the challenging L2 learning process.

The IL2S, OL2S, and L2LE were also found to have a significant effect on English achievement. This finding is in accord with the recent study (Zhao et al., 2022) reporting that L2 proficiency is positively influenced by L2LE and IL2S, but negatively impacted by OL2S. Similarly, the finding is also related to Papi’s study (2010) which revealed that OL2S significantly made L2 learners feel anxious, and the negative effect of anxiousness as a negative emotion on academic performance has been confirmed by several previous studies (e.g., MacIntyre, 1999; Gregersen and Horwitz, 2002; Horwitz, 2010; Dewaele and MacIntyre, 2014).

With regard to the pathways mediated by the three dimensions of L2MSS, in comparison to each other, the pathway of FLE → L2LE → EA had the largest effect size, followed by FLE → IL2S → EA; the pathway of OL2S may regress the positive effect of FLE on English achievement. It is true that learning enjoyment can positively and directly predict academic performance, but relying solely on this direct connection to improve foreign language performance might not be effective enough; then if the mediation role could serve as an entry point, it may aggregate direct and indirect effectiveness (Dong and Liu, 2022). Therefore, in foreign language teaching, in addition to the pivotal effects of a pleasant foreign language learning atmosphere and improving students’ FLE, more attention needs to be paid to the learners’ past EFL learning experiences and factors that stimulate their intrinsic motivation. Similarly, appropriately reducing the negative impact of external motivation will also be conducive to achieving better academic performance.

## 6. Limitations and future research

Although the research goals were achieved in the present study, there are still some limitations that may direct future research. Firstly, the convenience sampling suffered from a lack of diversity. In spite of involving students at different English proficiency levels, our participants were recruited from a single institution, which is one of the prestigious universities in China, so the overall English proficiency level of the students is higher than average. Therefore, we are cautious to generalize the finding to different educational contexts since the sample in the present study could not represent the whole EFL population. Studies on diverse populations in different foreign language contexts, and of a wide range of demographic and educational backgrounds are needed to verify the existing results. Secondly, only the quantitative method was employed in the present study. Hence, the intrinsic reasons and individual differences in the interconnection of variables were not investigated in depth. The mixed-methods approach is welcomed in future studies to gain a clearer and deeper understanding of the relationship between emotions, motivation, and L2 proficiency. Lastly, the cross-sectional design of the present study makes it fail to capture the changes in the variables and their relationship during the whole tertiary education. A portfolio of students’ emotions, motivation, and L2 achievement across time is expected in future research.

## 7. Pedagogical implications

Despite the limitations, the findings of the current study have some pedagogical implications for tertiary EFL education. These findings in line with previous studies confirm that teachers are highly conducive to bringing learning enjoyment to students (e.g., Dewaele and MacIntyre, 2016; Jin and Zhang, 2018; Dewaele et al., 2019b; Li et al., 2019; Alrabai, 2022), and the close ties between classroom emotions and motivation (e.g., Jin and Zhang, 2018; Li et al., 2019; Zhang et al., 2020; Pan and Zhang, 2021). FLE is crucial because it makes it possible to study English by enabling students to maximize their capacity for quick and thorough comprehension. Therefore, FLE must be viewed by EFL teachers as one of their top priorities in the classrooms (Ahmadi-Azad et al., 2020). Positive personality traits of the teacher, such as openness, extroversion, and agreeableness, are proven to be prominent factors (Ahmadi-Azad et al., 2020). Accordingly, the L2 learning process will be improved by instructors who are encouraging, supportive, nice, funny, optimistic, well-organized, competent, and considerate, and such teachers tend to put students in a positive environment and a good learning atmosphere (Arnold, 1999, 2011; Dewaele and MacIntyre, 2014; Dewaele et al., 2017, 2019b; Li, 2019; Dewaele and Li, 2021). Additionally, how teachers relate with their students is very important (Dewaele and MacIntyre, 2016; Moskowitz and Dewaele, 2021). Teachers’ encouragement and recognition can significantly boost students’, especially unsuccessful students’ sense of accomplishment and, consequently, stimulate students’ motivation for learning. In short, the strong connection between enjoyment, through motivation, and then academic achievement forms an effective path to creating a positive learning environment for students, stimulating latent motivation, and facilitating the learning process to achieve better learning outcomes.

## 8. Conclusion

The current study examined the connection between two crucial variables in EFL learning, namely FLE and L2 motivation, and their contribution to Chinese tertiary EFL learners’ English achievement at different proficiency levels. Overall, FLE and L2 motivation increase as the English proficiency level develops. College students enjoy higher levels of FLE than secondary school students. The greatest variation in FLE across English proficiency levels lies in learners’ internal factors while FLE due to external factors of teachers and atmosphere varied less. Chinese tertiary EFL learners were more stimulated by intrinsic motivation, which differs significantly across English proficiency levels, while extrinsic factors in L2MSS were less motivationally driven and did not vary significantly across different language proficiency levels. Moreover, FLE positively predicts L2 motivation of learners at different language proficiency levels, although the strength differs. The L2 motivation of learners at the elementary proficiency level was more likely to be influenced by external factors such as an enjoyable learning atmosphere, whereas students at the advanced proficiency level were more likely to be motivated by the pleasant feelings brought about by the teacher and themselves. EFL learners’ FLE could significantly and positively influence their academic achievement, and the L2 motivation had a partial mediation effect on the connection between FLE and English achievement. Therefore, the role of motivation can be fully utilized to facilitate FLE and work with FLE synergistically to help EFL learners achieve better learning outcomes through the path of enjoyment to motivation and motivation to academic achievement. This study supports the findings of related research in other contexts, and broadens and deepens the understanding of the contribution of positive emotions and motivation in language learning, thereby throwing light on English teaching and learning in tertiary education in China.

## Data availability statement

The raw data supporting the conclusions of this article will be made available by the authors, without undue reservation.

## Ethics statement

Ethical approval was not provided for this study on human participants because upon administering the questionnaire, the participants were reassured to participate in the survey voluntarily and informed of the purpose of this anonymous data collection. Written informed consent for participation was not required for this study in accordance with the national legislation and the institutional requirements.

## Author contributions

HW and LX conceptualized, designed the study, and drafted the manuscript. HW and JL collected the data and processed the data. All authors revised the manuscript and approved the submitted version.

## Funding

This research was supported by the Social Science Foundation of Liaoning Province (grant no. L21AYY002).

## Conflict of interest

The authors declare that the research was conducted in the absence of any commercial or financial relationships that could be construed as a potential conflict of interest.

## Publisher’s note

All claims expressed in this article are solely those of the authors and do not necessarily represent those of their affiliated organizations, or those of the publisher, the editors and the reviewers. Any product that may be evaluated in this article, or claim that may be made by its manufacturer, is not guaranteed or endorsed by the publisher.

## Footnotes

^1^The formula is from the official websites of CET-4 in the National Education Examinations Authority: https://cet.neea.edu.cn/html1/folder/19081/5124-1.htm.
