# Supplementary material for: Connecting foreign language enjoyment and English proficiency levels: The mediating role of L2 motivation
Source: Front Psychol. 2023 Feb 9;14:1054657. doi: 10.3389/fpsyg.2023.1054657 (PMC9948615; doi:10.3389/fpsyg.2023.1054657)
Supplement: Supplementary file 1 [file Questionnaire.DOCX]

**Questionnaire on FLE and L2 Motivation**

**Background Information**

Gender: ______________

Grade: _______________

CET score: ________/710

| 1. 我享受英语学习。 | I enjoy learning English. |
| --- | --- |
| 1. 学英语的过程中，我学了很多有趣的事情。 | I’ve learnt interesting things. |
| 1. 在班里，我为自己的英语成绩感到自豪。 | In class, I feel proud of my achievement. |
| 1. 老师总是鼓励我们。 | The teacher is encouraging. |
| 1. 老师很友善。 | The teacher is friendly. |
| 1. 我喜欢英语老师（包括他\她的语音、语调和教学风格），所以我对英语课很感兴趣。 | I like the English teacher (e. g. his/her pronunciation, intonation, and teaching style), so I am interested in English class. |
| 1. 英语课堂气氛活跃。 | The English classroom atmosphere is lively. |
| 1. 我身边有很好的英语课堂学习氛围。 | There is a good learning atmosphere. |
| 1. 我喜欢课堂上通过师生、生生互动的方式来学习英语。 | I like learning English through teacher-student and student-student interaction in the classroom. |
| 1. 我会想象自己在流畅地阅读英文报刊或者网站。 | I can imagine myself reading English newspapers or websites fluently. |
| 1. 我会想象自己听懂英语新闻、看懂英文影视剧。 | I can imagine myself understanding English news, English movies, and TV dramas. |
| 1. 我会想象自己与国际友人用英语流利地交谈。 | I can imagine myself living abroad and using English effectively in communication with the locals. |
| 1. 我会想象自己在职业生涯中使用英语交流。 | 13. I would imagine myself communicating in English during my career. |
| 1. 我会想象自己是同龄人中英语学得很好的学霸。 | I would imagine that I am the best student among my peers who learns English well. |
| 1. 我会想象自己在英语考试中顺利答题并取得高分。 | I can imagine myself passing the English test with a high score. |
| 1. 英语学习很重要，因为我周围的人希望我学英语。 | Learning English is necessary because people surrounding me expect me to do so. |
| 1. 英语学习很重要，因为我尊敬的人认为我应该学英语。 | I consider learning English important because the people I respect think that I should do that. |
| 1. 英语学习很重要，因为和我要好的朋友觉得学英语很重要。 | English learning is important because my close friends think it is important to learn English. |
| 1. 如果我英语不好，别人会认为我是个差生。 | If my English is not good, people will think that I am a poor student. |
| 1. 如果在英语考试中得分低，我会觉得很丢脸。 | I would feel humiliated if I scored low on the English test. |
| 1. 英语学习很重要，因为大家普遍认为受过良好教育的人就应该英语好。 | English learning is important because it is widely believed that well-educated people should be good at English. |
| 1. 我会主动从不同的渠道学习英语，把握每一个学习英语的机会。 | I will take the initiative to learn English from different channels and seize every opportunity to learn English. |
| 1. 我觉得学英语很有趣。 | I find learning English really interesting. |
| 1. 我感觉学英语时，时间过得很快。 | I think time passes faster while learning English. |
| 1. 我总是期待上英语课。 | I always look forward to English classes. |
| 1. 我想在学校有更多的英语课。 | I’d like to have more English classes at school. |
| 1. 我一直都很喜欢学英语。 | I have always enjoyed learning English. |
